# Supplementary material for: Positive charge in the K-loop of the kinesin-3 motor KIF1A regulates superprocessivity by enhancing microtubule affinity in the one-head–bound state
Source: J Biol Chem. 2022 Dec 20;299(2):102818. doi: 10.1016/j.jbc.2022.102818 (PMC9871336; doi:10.1016/j.jbc.2022.102818)
Supplement: Supporting information [file mmc1.pdf]

Positive charge in the K-loop of the kinesin-3 motor KIF1A regulates superprocessivity by enhancing microtubule affinity in the one-head-bound state

Taylor M. Zaniewski and William O. Hancock

Departments of Chemistry and Biomedical Engineering, Pennsylvania State University, University Park, Pennsylvania, USA

Supporting Information

Figures S1-S5.

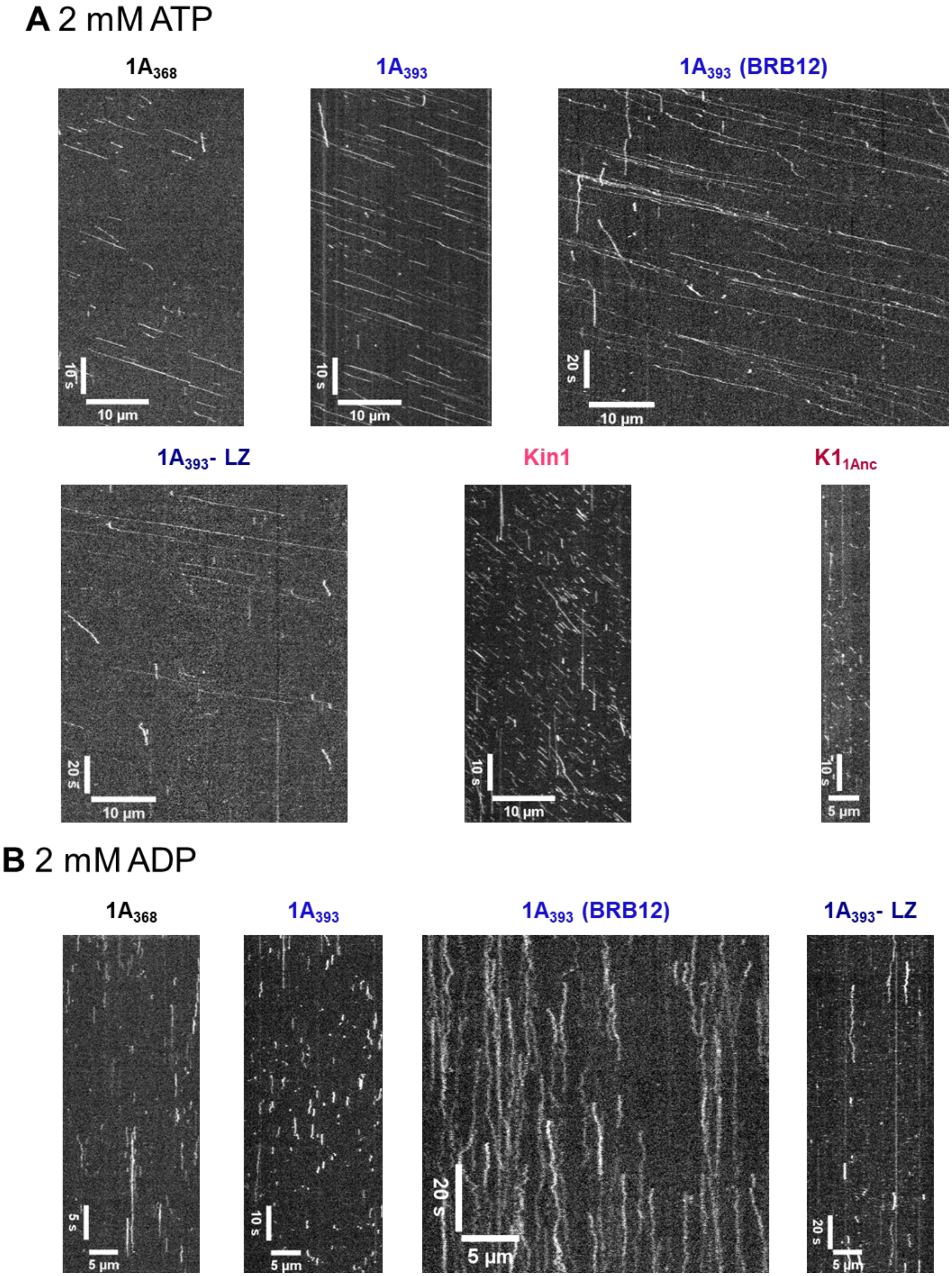

Figure S1: **Example kymographs for different KIF1A and Kin1 constructs in ATP and ADP**
A, Kymograph of each construct in 2 mM ATP and BRB80 (unless otherwise noted). Note that the $1A_{393}$ panel is reproduced from Fig. 1B in the main text and shown here for comparison to other constructs.

B, Kymograph of each construct in 2 mM ADP and BRB80 (unless otherwise noted). Individual kymographs were analyzed from videos at various frame rates; scale bars reflect this distinction.

	Domain	Sequence	Total Charges	Net Charge at pH 6.9	pI
Dimerization Domains	**LZ**	VKQLEDKVEELASKNYHLENEVARLKKLVG	(+7, -6)	+ 0.2	7.2
	**LZ + Linker**	GAGVKQLEDKVEELASKNYHLENEVARLKKLV<br>GVPRAMLVPRGPVHRLGDPPVAT	(+11, -7)	+ 2.5	9.3
	**K560**<br>(345-560)	AEEWKRRYEKEKEKNARLKGKVEKLEIELARW<br>RAGETVKAEEQINMEDLMEASTPNLEVEAAQT<br>AAAEAALAAQRTALANMSASVAVNEQARLATE<br>CERLYQQLDDKDEEINQQSQYAEQLKEQVMEQ<br>EELIANARREYETLQSEMARIQQENESAKEEV<br>KEVLQALEELTVNYDQKSQEIDNKNKDIDALN<br>EELQQKQSVFNAASTELQQLKDMS	(+28, -49)	- 21	4.5
	**KIF1A CC1**<br>(394-523)	MTNALVGMSPSSSLSALSSRAASVSSLHERIL<br>FAPGSEEAIERLKETEKIIAELNETWEEKLRR<br>TEAIRMEREALLAEMGVAMREDGGTLGVFSPK<br>KTPHLVNLNEDPLMSECLLYYIKDGVTRVGRE<br>DA	(+16, -22)	- 5.5	5
Neck Coil Domains	**Kin1 Neck Coil**<br>(345-405)	AEEWKRRYEKEKEKNARLKGKVEKLEIELARW<br>RAGETVKAEEQINMEDLMEASTPNLEVEAS	(+13, -16)	- 3.0	5.0
	**KIF1A Neck Coil**<br>(369-393)	IRELKDEVTRLRDLLYAQGLGDITD	(+4, -6)	- 2.1	4.7
	**KIF1A NC+CC1**<br>(369-523)	IRELKDEVTRLRDLLYAQGLGDITDMTNALVG<br>MSPSSSLSALSSRAASVSSLHERILFAPGSEE<br>AIERLKETEKIIAELNETWEEKLRRTEAIRME<br>REALLAEMGVAMREDGGTLGVFSPKKTPHLVN<br>LNEDPLMSECLLYYIKDGVTRVGREDA	(+20, -28)	- 7.4	5.0

Figure S2: **Charge of the neck coil domains impacts motor properties of KIF1A**
A, Table of the different dimerization and neck coil domains used in this study with the sequence, total number of charged residues, net charge of the domain at pH 6.9 and the pI. Net charge and pI were calculated using (http://protcalc.sourceforge.net/)

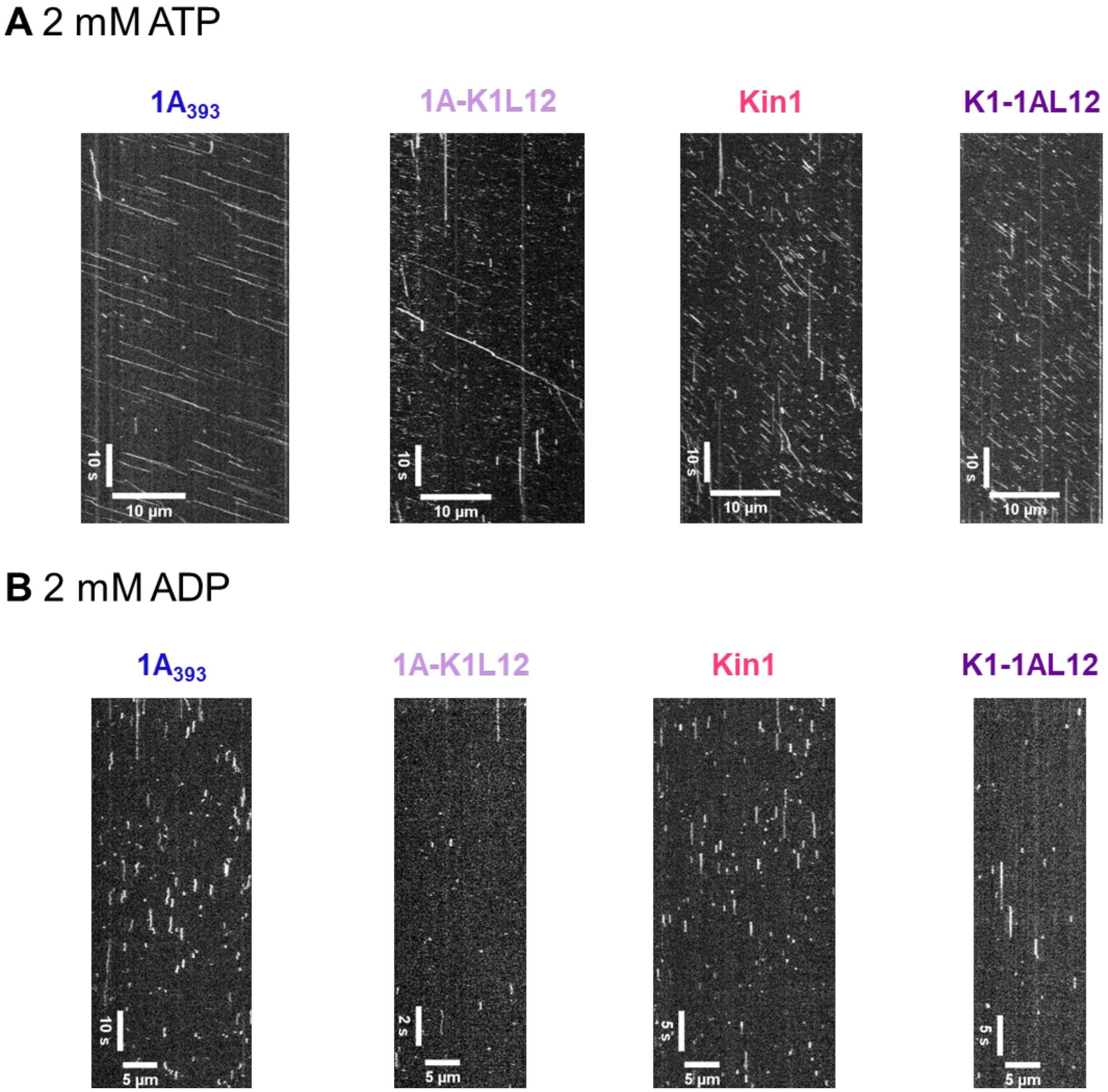

Figure S3: **Example kymographs of constructs in ATP and ADP**
A, Raw kymograph data for different constructs used in Fig. 2 in 2 mM ATP and BRB80. Note that the 1A393 panel is reproduced from Fig. S1A, and is shown here for comparison between constructs. **B**, Raw kymograph data of different constructs used in Fig. 2 dwell time measurements in 2 mM ADP and BRB80.

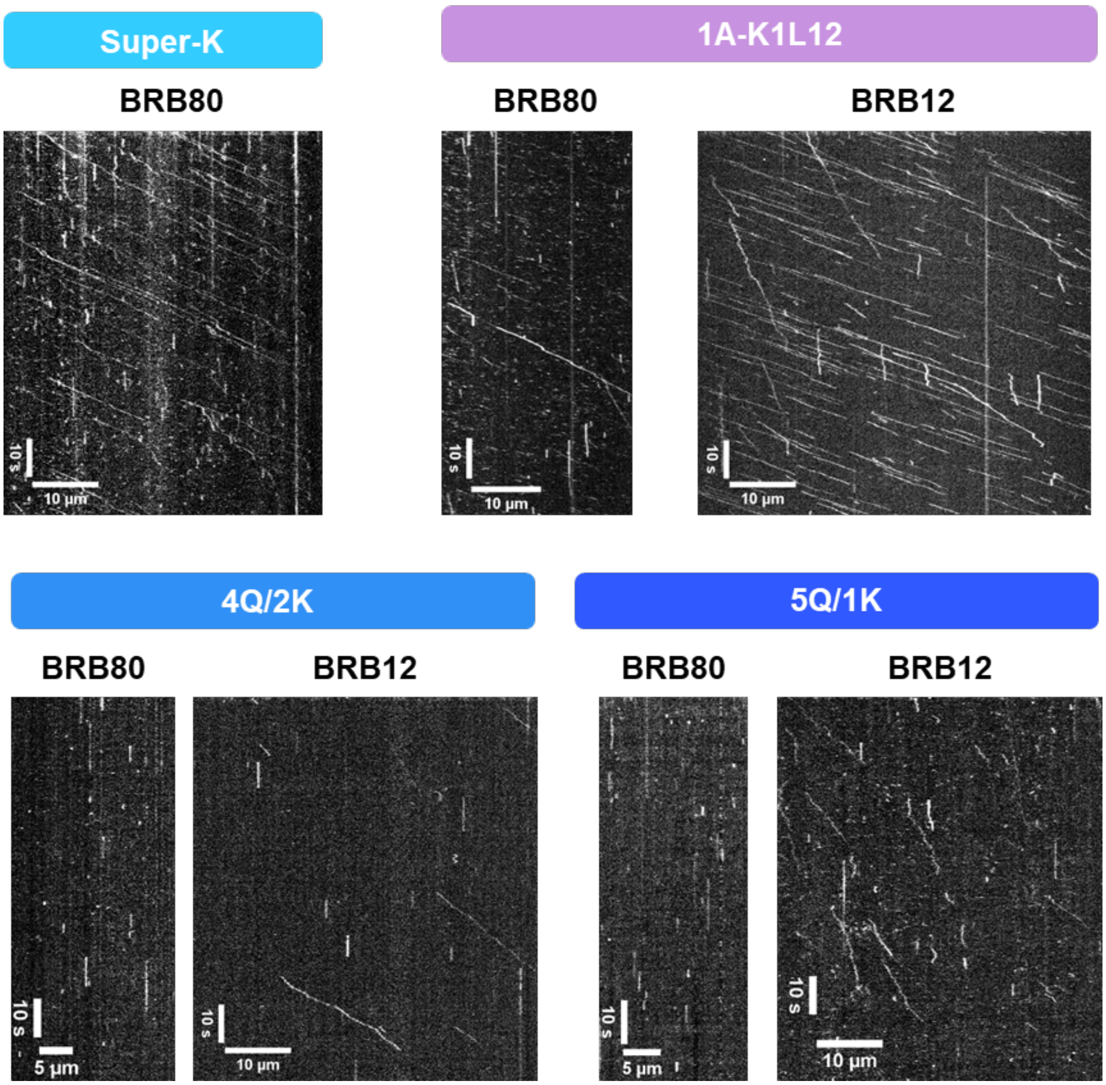

Figure S4: **Example Kymographs of Charge Mutants in BRB80 and BRB12**
Representative raw kymograph data for the velocity and run length data presented in Figure 4. Note that the 1A-K1L12 BRB80 panel is reproduced from panel in Fig. S3A and shown for comparison purposes.

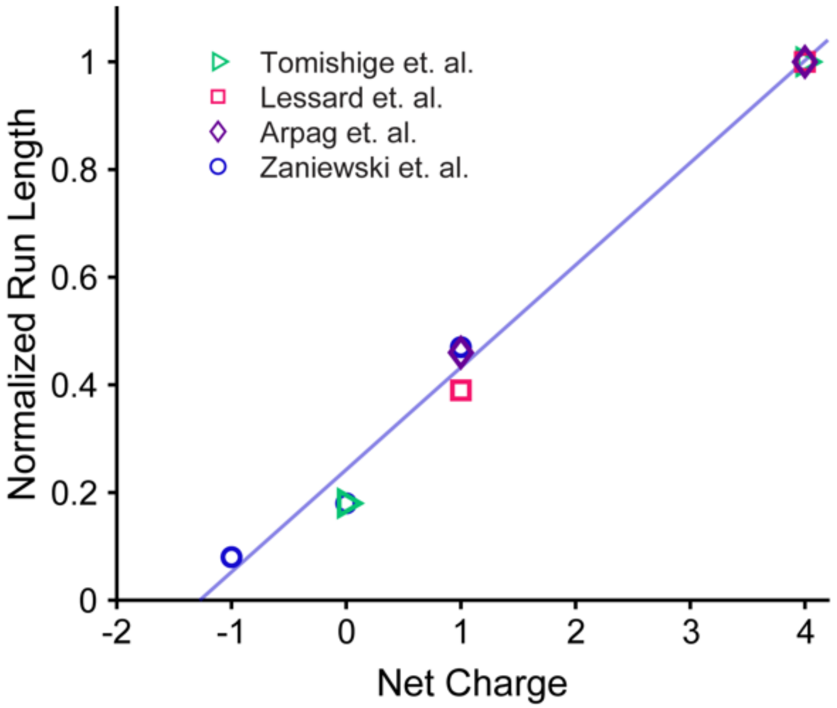

	Construct	K-Loop Charge	RL (µm)	RL (norm)
Tomishige *et. al.* [30]	Unc104-WT	4	9.5	1
	Unc104-Swap	0	1.8	0.2
Lessard *et. al.* [27]	1A-WT-LZ	4	6.2	1
	1A-3Ala-LZ	1	2.4	0.4
Arpag *et. al.* [19]	1A-WT-LZ	4	7.9	1
	1A-Swap-LZ	1	3.6	0.5
Zaniewski *et. al.* *(this study)*	$1A_{393}$	4	14.3	1
	1AK1L12	1	6.7	0.5
	1A-4Q	0	2.6	0.2
	1A-5Q	-1	1.2	0.1

Figure S5: **Published run lengths versus K-loop charge for stably dimerized KIF1A constructs in BRB12**

(Top) Plot of normalized run length versus net charge of Loop-12 for the present study and three published studies. Run lengths are normalized to the respective wild-type value (+4 net charge) for each study. Line represents fit to the relative run length versus charge for the present study (see Figure 6 for corresponding plot of non-normalized data). All experiments were performed in 12 mM PIPES buffer using constitutively active KIF1A dimers stabilized by an added coiled-coil domain. (Bottom) Actual and normalized run lengths from the four studies.
